# Self-Reported Parental Exposure to Pesticide during Pregnancy and Birth Outcomes: The MecoExpo Cohort Study

**DOI:** 10.1371/journal.pone.0099090

**Published:** 2014-06-20

**Authors:** Flora Mayhoub, Thierry Berton, Véronique Bach, Karine Tack, Caroline Deguines, Adeline Floch-Barneaud, Sophie Desmots, Erwan Stéphan-Blanchard, Karen Chardon

**Affiliations:** 1 Laboratoire PériTox, Unité mixte Université – INERIS (EA 4285-UMI 01), Université de Picardie Jules Verne, Amiens, France; 2 Unité NOVA, Institut National de l'Environnement Industriel et des Risques, Verneuil en Halatte, France; 3 Médecine Néonatale, Pôle Femme-Couple-Enfant, Centre Hospitalier Universitaire d'Amiens, Amiens, France; 4 Unité ISAE, Institut National de l'Environnement Industriel et des Risques, Verneuil en Halatte, France; 5 Unité TOXI, Institut National de l'Environnement Industriel et des Risques, Verneuil en Halatte, France; 6 Faculty of Medicine, Tishreen University, Latakia, Syria; Geisel School of Medicine at Dartmouth College, United States of America

## Abstract

The MecoExpo study was performed in the Picardy region of northern France, in order to investigate the putative relationship between parental exposures to pesticides (as reported by the mother) on one hand and neonatal parameters on the other. The cohort comprised 993 mother-newborn pairs. Each mother completed a questionnaire that probed occupational, domestic, environmental and dietary sources of parental exposure to pesticides during her pregnancy. Multivariate regression analyses were then used to test for associations between the characteristics of parental pesticide exposure during pregnancy and the corresponding birth outcomes. Maternal occupational exposure was associated with an elevated risk of low birth weight (odds ratio (OR) [95% confidence interval]: 4.2 [1.2, 15.4]). Paternal occupational exposure to pesticides was associated with a lower than average gestational age at birth (−0.7 weeks; p = 0.0002) and an elevated risk of prematurity (OR: 3.7 [1.4, 9.7]). Levels of domestic exposure to veterinary antiparasitics and to pesticides for indoor plants were both associated with a low birth weight (−70 g; p = 0.02 and −160 g; p = 0.005, respectively). Babies born to women living in urban areas had a lower birth length and a higher risk of low birth length (−0.4 cm, p = 0.006 and OR: 2.9 [1.5, 5.5], respectively). The present study results mainly demonstrate a negative correlation between fetal development on one hand and parental occupational and domestic exposure to pesticides on the other. Our study highlights the need to perform a global and detailed screening of all potential physiological effects when assessing in utero exposure to pesticides.

## Introduction

Human exposure to pesticides is a very complex phenomenon, since it involves many different compounds, sources of exposure and exposure pathways (i.e. respiratory, cutaneous and intestinal pathways). Once pesticides have been applied, the primary compounds and their degradation products are dispersed into the air, water and soil. Human exposure to pesticides can be occupational (through agriculture, floristry, municipal maintenance, etc.), dietary (through the consumption of food contaminated by pesticide residues), domestic (through the spraying of houseplants or garden plants, the eradication of domestic insect pests (such as mosquitoes, flies, etc.) and the use of antiparasitics in humans or in domestic pets) or environmental (i.e. the inhalation of volatilized pesticides of agricultural or non-agricultural origin) [1]–[2]. Although occupational exposure has been extensively investigated, there are few studies of domestic exposure [2].

Pesticide exposure during pregnancy is becoming an increasingly important public health issue because it may affect the development of the exposed fetus. The association between pesticide exposure in pregnant women and fetal growth has [11], [14]. However, this topic is still subject to debate because the various studies did not reach consistent conclusions - probably because of differences in location, exposure assessment methods and the type and number of compounds investigated [3]–[12]. Furthermore, most of these epidemiological studies focused on a very specific population, such as farmers or other populations with high levels of pesticide exposure (e.g. those living near to crop-farming areas or other areas with intensive pesticide use). To the best of our knowledge, very few studies [9], [10], [13], [14] have studied the relationship between “routine” domestic/dietary parental exposure on one hand and birth outcomes on the other. There are few data on the need for prevention of these types of exposure, and these data can only be gathered in general population cohorts.

The primary objective of the present MecoExpo study was to assess the different types of prenatal exposure to pesticides in the Picardy region of northern France (a region which is characterized by a high birth rate (13.1‰, according to the French National Institute of Statistics and Economic Studies (*Institut National de la Statistique et des Études Économiques*), relative to the national average. A secondary objective was to investigate the relationship between the different modes of exposure on one hand and birth outcomes on the other. Prenatal exposure to pesticides was assessed via a self-questionnaire filled out by the mother; this is the only method that can simultaneously gather information on all the various sources of intrauterine exposure to pesticides (i.e. occupational, domestic, environmental and dietary exposure) in a sample of the general population.

## Methods

### 2.1 Study participants

The MecoExpo cohort (comprising 993 mother-newborn pairs) was recruited between January 2011 and January 2012. Eleven of Picardy's 16 maternity clinics agreed to participate in the study. Unfortunately, the region's neonatal intensive care units (which treat newborns suffering from a severe neonatal disease or with a gestational age <32 weeks at birth) did not participate.

To be included in the MecoExpo cohort, the mother had to be had to be (i) 18 years of age or older and (ii) fluent enough in French to understand the study's objectives and procedures. Mothers aged under 18 and mothers who did not have full parental authority over their child (such as incarcerated persons) were excluded from the study. Multiple births were also excluded from the present study.

The study and both maternal and neonatal data collection (questionnaire, medical records) were approved by the local investigational review board (*Comité de Protection des Personnes dans la Recherche Biomédicale de Picardie*). Potential participants were given a verbal presentation of the study by their attending pediatrician in the maternity clinic. All mothers signed an informed consent giving us permission to enroll them and their infants in the study. A study information sheet was given to the women prior to their completion of the study questionnaire. During completion of the self-administred questionnaire, an investigating physician was always on hand to provide explanations but did not influence the answers. In view of the many different aspects of possible exposure probed by the questionnaire and in order to shorten and facilitate the questionnaire's completion, the respondee was instructed not to answer a given question if in any doubt. Most of the questions were “yes”/“no” or multiple choice questions. Open questions were used to gather additional information, when necessary. None of the data collected during the study enabled the direct identification of participants.

### 2.2 Characteristics of intrauterine exposure to pesticides in a questionnaire-based survey

Mothers from the cohort completed the questionnaire during their stay in the maternity clinic (4 to 5 days after giving birth). The questionnaire addressed occupational, domestic, environmental and dietary sources of pesticide exposure. Prior to use in the study, the questionnaire was validated in a sample of 25 women (aged between 25 and 45 and from various socioprofessional categories). It was then modified by taking account of the women's answers, comments and questions. The final questionnaire comprised 44 items and took respondees about 15 minutes to read and complete.

#### 2.2.1 Occupational exposure to pesticides

The mother was asked to state (i) whether she had ever worked during the pregnancy (and, if so, the duration), (ii) whether her occupation and/or that of the father involved exposure to pesticides (e.g. in agriculture, animal husbandry, gardening, etc.) and (iii) the modalities of maternal exposure to pesticides (the duration and location of exposure, the type of compound and the use or not of protective equipment).

#### 2.2.2 Domestic exposure to pesticides

The questionnaire probed possible uses of pesticides in the mother's home during her pregnancy (whether applied directly by the mother or indirectly by another person). The mother was asked to report use of (i) antiparasitics for human administration (to treat lice, scabies, ticks, etc.), (ii) antiparasitics for domestic pets (to treat fleas, ticks, etc.) and the number and type of treated animals, (iii) insecticides, herbicides, fungicides and plant growth regulators for treating houseplants or garden plants and (iv) pesticides for eradicating domestic insect pests (such as flies, mosquitoes, etc.).

#### 2.2.3 Environmental exposure to pesticides

The mother was asked to state whether she lived within 1 km of (i) green areas (a park, a sports field, etc.), (ii) a crop field, (iii) a highway, a railway or an airport. The mother was also asked to state the locality in which she had lived during her pregnancy. We then queried a database (produced by the French National Institute of Statistics and Economic Studies) to obtain the population density for each locality. The localities were then arbitrarily divided into rural localities (<2 000 inhabitants/km^2^) and urban localities (≥2 000 inhabitants/km^2^).

#### 2.2.4 Questions on dietary exposure

The mother was asked to state (i) her total dietary intake of the main food categories (fruits and vegetables, cereals, milk products and meat) during pregnancy (possible answers were “never or almost never”, “not every day”, “every day”, “several times a day”) and (ii) the frequency with which the consumed fruits and vegetables, milk products and meat were organic foods (with possible answers of “never”, “sometimes”, “most of the time ”).

### 2.3 Birth outcomes

Neonatal data - gestational age (weeks of amenorrhea), birth weight (g), birth length (cm) and head circumference at birth (cm) - were extracted from medical records by the maternity clinic's pediatrician. Prematurity was defined as birth before 37 weeks of amenorrhea. Low birth weight or length was defined as a birth weight or length (adjusted for the mother's age, weight, and height, the rank of pregnancy, the gestational age and the baby's gender) below the 5^th^ percentile (relative to normative data for France) [15]–[16]. Small head circumference was defined as a measurement (adjusted for gestational age and the baby's gender) below the 5^th^ percentile (relative to normative data for France) [15]. Infants with missing neonatal or maternal data on adverse birth outcomes were not considered in these analyses.

### 2.4 Confounding factors

Factors known or suspected (on the basis of the scientific literature) to have an effect on fetal development were considered as confounding factors, i.e. the mother's age and body mass index (BMI), parity, diabetes, hypertension, tobacco use, alcohol use, drug abuse, socioprofessional category (educational level and type of work), and the baby's gender.

### 2.5 Data processing

The study data (questionnaire data, birth outcomes and covariates) were recorded using Sphinx plus^2^ software (version 5.1.0.6, Le Sphinx Développement, Chavanod, France). Some questionnaire answers were combined, in order to obtain new, composite variables that were more relevant than those obtained from single questions. Thus, the composite variables used in the assessment of intrauterine exposure to pesticides included maternal or paternal occupational exposure, maternal domestic exposure to human antiparasitics, veterinary antiparasitics, and domestic pesticides for houseplants or garden plants, environmental exposure (the home's proximity to a crop fields, green areas, highways, railways or airports) and dietary exposure (consumption of fruits and vegetables, cereals, milk products and meat; consumption of organic fruits and vegetables, etc.).

### 2.6 Statistical analyses

The MecoExpo cohort's demographics and intrauterine exposure to pesticides were first characterized in a descriptive analysis. Quantitative parameters (gestational age, birth weight, etc.) were expressed as the mean and the standard deviation (SD). Qualitative parameters (prematurity, low birth weight, maternal occupational exposure to pesticides, etc.) were expressed as the number and the percentage of the study population.

Multivariate linear regression and logistic regression analyses were used to study the putative associations between birth outcomes and the characteristics of *in utero* exposure to pesticides. Using forward selection, covariates with a p-value<0.20 in a univariate analysis were fed into in the multivariate analyses. The risk of an adverse birth outcome (prematurity, small head circumference, and low birth weight and low birth length) was expressed as an odds ratio (OR) [95% confidence interval (CI)]. The 95% CI was calculated according to Woolf's method, with an alpha risk of 0.05. All statistical analyses were performed with SPSS software (V.20.0, Chicago, IL, USA).

## Results

### 3.1 Characteristics of the study population

A total of 993 mother-newborn pairs were included in the MecoExpo cohort. Given that some questionnaire answers or clinical values were missing, the total n for some variables was below 993 and so the corresponding missing data rates are also reported.

The characteristics of the MecoExpo study population are summarized in Table 1 and Table 2. The mean (SD) maternal age was 29 (5.2) and the mean pre-pregnancy BMI was 24.4 (5.5) kg/m^2^. Six percent of the mothers were diabetic and less than 5% had arterial hypertension during pregnancy. Forty-two percent of the women were primiparous, 46% had completed high school and 68% had been working during their pregnancy. About 30% of the mothers stated smoking during pregnancy; the corresponding values for drinking alcohol and illicit drug use were 3% and 1%, respectively. Overall, 86% of the women lived in rural localities (<2 000 inhabitants/km^2^).

**Table 1:** Maternal characteristics in the MecoExpo cohort (n = 993).

Variables	
Age (year)	Mean (SD)
	29 (5.2)
Parity:	n(%)
Primiparous	409 (41.7)
Multiparous	573 (58.3)
*Missing data*	*11*
Maternal diabetes during pregnancy:	n(%)
Yes	62 (6.2)
No	931 (93.8)
Maternal hypertension during pregnancy:	n(%)
Yes	47 (4.7)
No	946 (95.3)
Maternal smoking during pregnancy:	n(%)
Yes	293 (29.5)
No	700 (70.5)
Maternal educational level:	n(%)
≤High school completed	504 (53.7)
>High school completed	434 (46.3)
*Missing data*	*55*
Working during pregnancy:	n(%)
Yes	671 (67.6)
No	322 (32.4)
Place of residence during pregnancy:	n(%)
Rural locality	774 (85.9)
Urban locality	127 (14.1)
*Missing data*	*92*

**Table 2:** Newborn characteristics in the MecoExpo cohort (n = 993).

Variables	Mean (SD)	n (%)
Gestational age (weeks)	39.3 (1.3)	924 (93.1)
*Missing data*		*69 (6.9)*
Birth weight (g)	3340 (492)	932 (93.9)
*Missing data*		*61 (6.1)*
Birth length (cm)	49.5 (2.1)	922 (92.8)
*Missing data*		*71 (7.2)*
Head circumference at birth (cm)	34.4 (1.4)	911 (91.7)
*Missing data*		*82 (8.3)*
Gender:		
male		508 (51.2)
female		485 (48.8)
Prematurity:		
Yes		24 (2.6)
No		900 (97.4)
*Missing data*		*69*
Low birth weight:		
Yes		788 (94.9)
No		42 (5.1)
*Missing data*		*163*
Low birth length:		
Yes		56 (6.8)
No		764 (93.2)
*Missing data*		*173*
Small head circumference:		
Yes		34 (4.0)
No		822 (96.0)
*Missing data*		*137*

### 3.2 Characteristics of in utero exposure to pesticides

The main characteristics of prenatal exposure to pesticides (as assessed by the mothers' questionnaire data) are summarized in Table 3 and 4.

**Table 3:** Characteristics of prenatal exposure to pesticides (n = 993).

Variables	n (%)
**Occupational exposure to pesticides**	
Mother:	
Yes	43 (4.3)
No	950 (95.7)
Father:	
Yes	86 (8.7)
No	907 (91.3)
**Maternal domestic exposure to pesticides**	
Human antiparasitics:	
Yes	328 (33.0)
No	665 (67.0)
Veterinary antiparasitics:	
Yes	215 (21.7)
No	776 (78.3)
*Missing data*	*2*
Pesticides against insects:	
Yes	127 (13.0)
No	850 (87.0)
*Missing data*	*16*
Pesticides for indoor plants:	
Yes	37 (3.7)
No	956 (96.3)
Pesticides for outdoor plants:	
Yes	166 (17.0)
No	813 (83.0)
*Missing data*	*14*
**Maternal environmental exposure to pesticides**	
Proximity to a crop field (<1 km):	
Yes	527 (58.2)
No	378 (41.8)
*Missing data*	*88*
Proximity to a green area:	
Yes	707 (72.7)
No	266 (27.3)
*Missing data*	*20*
Proximity to a highway, railway or airport:	
Yes	366 (37.4)
No	612 (62.6)
*Missing data*	*15*

**Table 4:** Characteristics of prenatal exposure to pesticides (n = 993) (continued).

Variables	n (%)
**Maternal dietary exposure to pesticides (total food intake)**	
Fruits and vegetables:	
Never or almost never	18 (1.8)
Not every day	319 (32.3)
Every day	362 (36.6)
Several times a day	289 (29.3)
*Missing data*	*5*
Cereals:	
Never or almost never	4 (0.4)
Not every day	173 (17.6)
Every day	532 (54.1)
Several times a day	274 (27.9)
Missing data	10
Dairy products:	
Never or almost never	8 (0.8)
Not every day	111 (11.3)
Every day	430 (43.7)
Several times a day	436 (44.3)
*Missing data*	*8*
Meat:	
Never or almost never	23 (2.4)
Not every day	365 (37.5)
Every day	489 (50.2)
Several times a day	97 (10.0)
*Missing data*	*19*
**Maternal dietary exposure to pesticides (organic food intake)**	
Fruits and vegetables:	
Never	518 (52.4)
Sometimes	375 (38.0)
Most of the time	95 (9.6)
*Missing data*	*5*
Dairy products:	
Never	654 (66.3)
Sometimes	265 (26.9)
Most of the time	68 (6.9)
*Missing data*	*6*
Meat:	
Never	827 (84.0)
Sometimes	137 (13.9)
Most of the time	20 (2.0)
*Missing data*	*9*

#### 3.2.1 Occupational exposure to pesticides

43 mothers and 86 fathers (4.3% and 8.7% of the total sample, respectively) worked in an occupation in which there was potential for exposure to pesticides. Fifteen mothers and 42 fathers (1.5% and 4.2% of the total sample, respectively) had agricultural occupations.

#### 3.2.2 Domestic exposure to pesticides

33% of the mothers were exposed to human antiparasitics, with 22% exposed to veterinary antiparasitics, 13% exposed to domestic insecticides, 4% exposed to pesticides for houseplants and 17% exposed to pesticides for garden plants.

#### 3.2.3 Environmental exposure to pesticides

58% of the mothers lived near to a crop field crop, 73% lived near to a green area and 37% lived near to a highway, railroad line or airport.

#### 3.2.4 Dietary exposure to pesticides

29% of mothers consumed fruits and vegetables “several times” a day and 44% consumed milk products “several times a day”. Fifty percent consumed cereals “every day” and 50% consumed meat “every day”.

Only 10% of the mothers consumed organic fruits and vegetables “most of the time”, whereas the corresponding proportions organic milk products and organic meat were 7% and 2%, respectively.

### 3.3 Multivariate associations between fetal growth and estimated in utero exposure to pesticides

#### 3.3.1 Gestational age and prematurity (Table 5, 6)

**Table 5:** Multivariate associations between factors related to *in utero* exposure to pesticides and gestational age and prematurity.

Variable	Gestational age (weeks)		Prematurity	
	n	Mean (SD)	Cases/controls	OR [95% CI]
**Total sample**	924	-	24/900	-
**Occupational exposure**				
Mother:				
Yes	21	39.2 (1.4)	1/20	NA
No	903	39.5 (1.3)	23/880	Reference
Father:				
Yes	80	38.8 (1.7)**	6/74	3.7 [1.4, 9.7]*
No	844	39.5 (1.3)	18/826	Reference
**Maternal domestic exposure**				
Human antiparasitics:				
Yes	307	39.6 (1.2)	7/300	0.8 [0.3, 2.0]
No	617	39.4 (1.4)	17/600	Reference
Veterinary antiparasitics:				
Yes	201	39.4 (1.4)	6/195	1.2 [0.5, 3.1]
Non	721	39.5 (1.3)	18/703	Reference
*Missing data*	*2*		*0/2*	-
Pesticides against insects:				
Yes	115	39.6 (1.2)	3/112	1.0 [0.3, 3.4]
No	794	39.4 (1.3)	20/774	Reference
*Missing data*	*15*	-	*1/14*	-
Pesticides for indoor plants:				
Yes	35	39.4 (1.2)	1/34	NA
No	889	39.5 (1.3)	23/866	Reference
Pesticides for outdoor plants:				
Yes	156	39.5 (1.2)	2/154	NA
No	754	39.5 (1.3)	21/733	Reference
*Missing*	*14*	-	*1/13*	-
**Maternal environmental exposure**				
Proximity to a crop field:				
Yes	490	39.4 (1.4)	17/473	2.1 [0.8, 5.4]
No	356	39.6 (1.3)	6/350	Reference
*Missing data*	*78*	-	*1/77*	-
Proximity to a green area:				
Yes	660	39.5 (1.3)	19/641	1.8 [0.6, 5.3]
No	244	39.5 (1.3)	4/240	Reference
*Missing data*	*20*	-	*1/19*	-
Proximity to a highway, railway or airport:				
Yes	339	39.5 (1.3)	8/331	0.9 [0.4, 2.2]
No	571	39.5 (1.3)	15/556	Reference
*Missing data*	*14*	-	*1/13*	-

**Table 6:** Multivariate associations between factors related to *in utero* exposure to pesticides and gestational age and prematurity (continued).

Place of residence during pregnancy:				
Rural locality	714	39.4 (1.4)	21/693	Reference
Urban locality	123	39.6 (1.3)	1/122	NA
*Missing data*	*87*	-	*2/85*	-
**Maternal dietary exposure (total food intake)**				
Fruits and vegetables:				
Never or almost never	16	39.3(1.4)	0/16	NA
Not every day	298	39.5(1.3)	8/290	Reference
Every day	333	39.4(1.3)	10/323	1.1 [0.4, 2.9]
Several times a day	272	39.6(1.3)	6/266	0.8 [0.3, 2.4]
*Missing data*	*5*	*-*	*0/5*	*-*
Cereals:				
Never or almost never	4	39.7 (0.5)	0/3	NA
Not every day	162	39.4 (1.2)	3/159	Reference
Every day	488	39.5 (1.4)	15/473	1.7 [0.5, 5.9]
Several times a day	260	39.5(1.3)	6/254	1.3 [0.3, 5.1]
*Missing data*	79	-	0/79	-
Dairy products:				
Never or almost never	7	39.6 (0.7)	0/7	NA
Not every day	105	39.4 (1.1)	2/103	Reference
Every day	399	39.4 (1.3)	10/389	1.3 [0.3, 6.1]
Several times a day	405	39.5 (1.4)	12/393	1.6 [0.4, 7.1]
*Missing data*	*8*	-	*0/8*	-
Meat:				
Never or almost never	22	39.5 (2.0)	1/21	NA
Not every day	338	39.5 (1.2)	4/334	Reference
Every day	458	39.4 (1.4)	16/442	3.0 [1.0, 9.1]
Several times a day	88	39.5 (1.6)	3/85	3.0 [0.7, 13.4]
*Missing*	*87*	-	*0/87*	-
**Maternal dietary exposure (organic food intake)**				
Fruits and vegetables:				
Never	481	39.4 (1.4)	13/468	0.6 [0.2, 1.9]
Sometimes	347	39.6 (1.2)	7/340	0.5 [0.1, 1.6]
Most of the time	91	39.3 (1.5)	4/87	Reference
*Missing data*	*5*	-	*0/5*	-
Dairy products:				
Never	607	39.5 (1.3)	17/590	1.8 [0.2, 13.9]
Sometimes	247	39.5 (1.3)	6/241	1.6 [0.2, 13.3]
Most of the time	64	39.5 (1.3)	1/63	Reference
*Missing data*	*6*	-	*0/6*	
Meat:				
Never	773	39.5 (1.3)	21/752	Reference
Sometimes	124	39.5 (1.3)	2/122	NA
Most of the time	18	39.2 (1.4)	1/17	NA
*Missing data*	*78*	-	*0/78*	-

*p = 0.05;

**p = 0.01;

***p = 0.001.

NA: an odds ratio was not available because too few cases were observed (n<3).

Estimated paternal occupational exposure to pesticides was significantly associated with lower gestational age (−0.7 weeks, relative to infants whose parents were not occupationally exposed to pesticides; p = 0.006) and a higher risk of prematurity (OR [95% CI]: 3.7 [1.4, 9.7]; p = 0.02). Maternal exposure (whether occupational, domestic, environmental or dietary) was not significantly associated with either gestational age or the risk of prematurity.

#### 3.3.2 Birth weight (as a continuous variable) and low birth (Table 7, 8)

**Table 7:** Multivariate associations between (i) variables related to *in utero* exposure to pesticides, (ii) birth weight (BW, in g) and low birth weight (LBW).

Variables	BW (g)		LBW	
	n	Mean (SD)	Cases/controls	OR [95% CI]
**Total sample**	932		42/786	
**Occupational exposure**				
Mother:				
Yes	19	3177 (521)	3/14	4.2 [1.2, 15.4]**
No	913	3343 (491)	39/772	Reference
Father:				
Yes	83	3213 (517)	1/96	NA
No	849	3353 (488)	41/690	Reference
**Domestic exposure**				
Humans antiparasitic:				
Yes	309	3397 (477)*	11/241	0.8 [0.4, 1.6]
No	623	3312 (497)	31/545	Reference
Veterinary antiparasitic:				
Yes	206	3286 (520)*	10/177	1.1 [0.5, 2.3]
No	724	3358 (482)	31/608	Reference
*Missing data*	*2*	-	*1/1*	-
Pesticides against insects:				
Yes	120	3373 (472)	4/95	0.8 [0.3, 2.2]
No	796	3338 (493)	37/680	Reference
*Missing data*	*16*	-	*1/11*	-
Pesticides for indoor plants:				
Yes	33	3186 (464)**	2/26	NA
No	899	3346 (492)	40/760	Reference
Pesticides for outdoor plants:				
Yes	156	3369 (479)	2/136	NA
No	762	3337 (492)	40/638	Reference
*Missing data*	14	-	0/12	-
**Environmental exposure**				
Proximity to a crop field:				
Yes	490	3328 (491)	25/411	1.2 [0.6, 2.4]
No	359	3352 (486)	15/304	Reference
*Missing data*	83	-	2/71	-
Proximity to a green area:				
Yes	663	3365 (498)	26/564	0.6 [0.3, 1.1]
No	250	3275 (470)	16/204	Reference
*Missing data*	*19*	-	*0/18*	-
Proximity to a highway, railway or airport:				
Yes	344	3380 (523)	15/259	0.9 [0.5, 1.8]
No	573	3317 (470)	27/484	Reference
*Missing data*	15	-	0/13	-

**Table 8:** Multivariate associations between (i) variables related to *in utero* exposure to pesticides, (ii) birth weight (BW, in g) and low birth weight (LBW) (continued).

**Dietary exposure (Total food intake)**				
Fruits and vegetables:				
Never or almost never	17	3152 (501)	2/13	Reference
Not every day	298	3316 (481)	16/250	0.4 [0.1, 2.0]
Every day	342	3329 (483)	11/289	0.3 [0.1, 1.3]
Several times a day	270	3388 (511)	13/230	0.4 [0.1, 1.8]
*Missing data*	5	-	0/4	-
Cereals:				
Never or almost never	4	3525 (527)	0/4	NA
Not every day	164	3360 (466)	7/141	Reference
Every day	495	3321 (489)	22/409	0.6 [0.3, 1.5]
Several times a day	259	3360 (508)	12/227	1.1 [0.4, 2.8]
*Missing data*	*71*	-	*1/5*	-
Dairy products:				
Never or almost never	8	3098 (431)	1/5	NA
Not every day	103	3251 (473)	8/86	Reference
Every day	408	3345 (475)	17/341	0.5 [0.1, 4.5]
Several times a day	405	3357 (510)	16/348	0.3 [0.0, 2.3]
*Missing data*	*8*		*0/6*	
Meat:				
Never or almost never	22	3275 (520)	1/20	NA
Not every day	340	3329 (465)	19/288	Reference
Every day	458	3332 (494)	19/388	0.7 [0.4, 1.4]
Several times a day	93	3393 (557)	3/78	0.6 [0.2, 2.0]
*Missing data*	*80*	-	*0/12*	-
**Dietary exposure (organic food intake)**				
Fruits and vegetables:				
Never	483	3304 (509)	23/399	1.6 [0.5, 5.4]
Sometimes	354	3399 (472)	16/300	1.5 [0.4, 5.2]
Most of the time	90	3283 (453)	3/83	Reference
*Missing data*	5	-	0/5	-
Dairy products:				
Never	617	3327 (505)	32/508	1.4 [0.7, 2.8]
Sometimes	245	3367 (469)	10/216	Reference
Most of the time	64	3346 (439)	0/4	NA
*Missing data*	6	-	0/6	-
Meat:				
Never	777	3329 (490)	39/657	Reference
Sometimes	126	3422 (492)	2/108	NA
Most of the time	20	3238 (518)	1/16	NA
*Missing data*	70	-	0/5	-

*P<0.05;

**P<0.01.

BW: birth weight; LBW: Low birth weight; NA: an odds ratio was not available because too few cases were observed (n<3).

Estimated maternal occupational exposure was associated with higher risk of low birth weight (OR [95% CI]: 4.2 [1.2, 15.4]; p = 0.01). Exposure to human and veterinary antiparasitics were both significantly associated with a lower birth weight (−70 g; p = 0.02 and −85 g; p = 0.04, respectively). Exposure to pesticides for houseplants was also associated with lower birth weight (−160 g; p = 0.03). No association with dietary exposure was observed.

#### 3.3.3 Birth length (as a continuous variable) and low birth length (Table 9, 10)

**Table 9:** Multivariate associations between (i) variables related to *in utero* exposure to pesticides, (ii) birth length (BL, in cm) and low birth length).

Variable	BL (cm)		LBL	
	n	Mean (SD)	Cases/controls	OR [95% CI]
Total sample	922		56/764	
**Occupational exposure**				
Mother:				
Yes	19	49.5 (1.9)	2/15	NA
No	903	49.5 (2.1)	54/749	Reference
Father:				
Yes	79	49.3 (2.0)	1/67	NA
No	843	49.5 (2.1)	55/697	Reference
**Domestic exposure**				
Humans antiparasitics:				
Yes	309	49.8 (2.0)	19/252	1.0 [0.6, 1.9]
No	613	49.3 (2.1)	37/512	Reference
Veterinary antiparasitics:				
Yes	201	49.3 (2.1)	9/173	0.7 [0.3, 1.4]
No	719	49.5 (2.1)	46/590	Reference
*Missing data*	*2*	-	*1/1*	-
Pesticides against insects:				
Yes	115	49.5 (1.9)	8/88	1.3 [0.6, 2.9]
No	793	49.5 (2.1)	46/666	Reference
*Missing data*	*14*	-	*2/10*	-
Pesticides for indoor plants:				
Yes	35	49.2 (2.0)	4/26	2.2 [0.7, 6.5]
No	887	49.5 (2.1)	52/738	Reference
Pesticides for outdoor plants:				
Yes	157	49.7 (2.2)	9/130	0.9 [0.4, 1.9]
No	752	49.4 (2.1)	47/623	Reference
*Missing data*	*13*	-	*0/11*	-
**Environmental exposure to pesticides**				
Proximity to a crop field (<1 km):				
Yes	491	49.6 (2.1)	32/404	1.0 [0.6, 1.8]
No	350	49.3 (2.0)	22/289	Reference
*Missing data*	*81*	-	*2/71*	-
Proximity to a green area:				
Yes	655	49.6 (2.2)	42/541	1.1 [0.6, 2.1]
No	249	49.2 (1.9)	14/206	Reference
*Missing data*	*18*	-	*0/17*	-
Proximity to a highway, railway or airport:				
Yes	339	49.6 (2.1)	23/276	1.2 [0.7, 2.9]
No	570	49.4 (2.1)	33/477	Reference
*Missing data*	*13*	-	*0/11*	-
Level of urbanization:				
Rural	726	49.5 (2.1)	34/606	Reference
Urban	119	49.1 (2.0)	15/93	2.9 [1.5, 5.5]*
*Missing data*	*77*	-	*7/65*	-

**Table 10:** Multivariate associations between (i) variables related to *in utero* exposure to pesticides, (ii) birth length (BL, in cm) and low birth length) (continued).

**Dietary exposure (total food intake)**				
Fruits and vegetables:				
Never or almost never	18	49.3 (2.2)	1/15	Reference
Not every day	301	49.4 (2.0)	19/253	1.1 [0.1, 9.0]
Every day	329	49.4 (2.2)	19/268	1.1 [0.1, 8.5]
Several times a day	269	49.7 (2.1)	17/224	1.1 [0.1, 9.2]
*Missing data*	*5*	-	*0/4*	-
Cereals:				
Never or almost never	4	50.2 (1.7)	0/4	NA
Not every day	159	49.4 (2.1)	11/132	Reference
Every day	493	49.4 (2.1)	27/402	0.8 [0.4, 1.7]
Several times a day	256	49.6 (2.2)	18/218	1.0 [0.5, 2.2]
*Missing data*	*81*	-	*0/8*	-
Dairy products:				
Never or almost never	7	48.1 (1.2)	0/5	NA
Not every day	105	49.3 (2.1)	8/87	Reference
Every day	396	49.4 (2.0)	21/328	0.7 [0.3, 1.6]
Several times a day	407	49.6 (2.2)	27/339	0.9 [0.4, 2.0]
*Missing data*	*7*	-	*0/5*	-
Meat:				
Never or almost never	22	49.3 (2.2)	0/20	NA
Not every day	338	49.4 (2.1)	25/279	Reference
Every day	457	49.5 (2.1)	24/382	0.7 [0.4, 1.3]
Several times a day	87	49.6 (2.3)	6/71	0.9 [0.4, 2.4]
*Missing data*	*89*	-	*1/12*	-
**Dietary exposure (organic food intake)**				
Fruits and vegetables:				
Never	484	49.3 (2.2)	36/388	1.5 [0.6, 4.0]
Sometimes	345	49.7 (1.9)	15/330	0.6 [0.3, 2.1]
Most of the time	88	49.4 (2.1)	5/83	Reference
*Missing data*	*5*	-	*0/4*	-
Dairy products:				
Never	611	49.4 (2.1)	39/498	1.1 [0.6, 2.0]
Sometimes	242	49.6 (2.0)	15/207	2.0 [0.4, 9.0]
Most of the time	62	49.8 (2.0)	2/55	Reference
*Missing data*	*3*	-	*0/4*	-
Meat:				
Never	769	49.4 (2.1)	47/642	Reference
Sometimes	126	49.6 (1.9)	7/102	0.9 [0.4, 2.1]
Most of the time	18	49.0 (2.1)	1/14	NA
*Missing data*	*80*	-	*1/6*	-

*P<0.05;

**P<0.01.

BL: birth length, LBL: low birth length. NA: an odds ratio was not available because too few cases were observed (n<3).

The risk of low of low birth length was significantly associated with the mother's residence in an urban area (OR [95% CI]: 2.9 [1.5, 5.5]; p = 0.01]. No associations with parental occupational exposure or dietary exposure were observed.

#### 3.3.4 Birth head circumference at birth (as a continuous variable) and small head circumference (Table 11, 12)

**Table 11:** Multivariate associations between (i) variables related to *in utero* exposure to pesticides, (ii) head circumference at birth (HCB, in cm) and (iii) small head circumference at birth.

Variable	HCB (cm)		SHCB	
	N	Mean (SD)	Cases/controls	OR [95% CI]
Total sample	911		34/822	
**Occupational exposure**				
Mother:				
Yes	21	34.5 (1.7)	2/36	NA
No	890	34.4 (1.4)	32/786	Reference
Father:				
Yes	79	34.2 (1.6)	4/70	1.3 [0.5, 4.2]
No	832	34.4 (1.4)	30/752	Reference
**Domestic exposure**				
Human antiparasitics:				
Yes	305	34.5 (1.4)	9/276	0.7 [0.3, 1.5]
No	606	34.3 (1.4)	25/546	Reference
Veterinary antiparasitics:				
Yes	193	34.3 (1.5)	8/175	1.1 [0.5, 2.6]
No	717	34.4 (1.4)	26/646	Reference
*Missing data*	*1*	-	*0/1*	-
Pesticides against insects:				
Yes	115	34.4 (1.3)	7/99	1.9 [0.8, 4.4]
Non	781	34.4 (1.4)	27/709	Reference
*Missing data*	*15*	-	*0/14*	-
Pesticides for indoor plants:				
Yes	33	34.2 (1.4)	2/29	NA
No	878	34.4 (1.4)	32/793	Reference
Pesticides for outdoor plants:				
Yes	149	34.6 (1.4)	8/135	1.5 [0.7, 3.5]
No	748	34.4 (1.4)	26/674	Reference
*Missing data*	*14*	-	*0/13*	-
**Environmental exposure**				
Proximity to a crop field:				
Yes	487	34.4 (1.4)	20/437	1.3 [0.6, 2.8]
No	345	34.4 (1.4)	11/314	Reference
*Missing data*	*79*	-	*3/71*	-
Proximity to a green area:				
Yes	644	34.5 (1.4)	21/586	0.7 [0.3, 1.3]
No	249	34.2 (1.4)	12/219	Reference
*Missing data*	*18*	-	*1/17*	-
Proximity to a highway, railway or airport:				
Yes	332	34.4 (1.4)	12/301	0.9 [0.5, 1.9]
No	565	34.5 (1.4)	22/508	Reference
*Missing data*	*14*	-	*0/13*	-

**Table 12:** Multivariate associations between (i) variables related to *in utero* exposure to pesticides, (ii) head circumference at birth (HCB, in cm) and (iii) small head circumference at birth (continued).

Place of residence during pregnancy:				
Rural locality	722	34.4 (1.4)	24/649	Reference
Urban locality	114	34.4 (1.5)	9/102	2.4 [1.1, 5.3]
*Missing data*	*75*	-	*1/71*	**-**
**Dietary exposure (total food intake)**				
Fruits and vegetables:				]
Never or almost never	18	34.0 (1.3)	0/16	NA
Not every day	292	34.4 (1.4)	16/263	Reference
Every day	334	34.4 (1.4)	11/297	0.6 [0.3, 1.3]
Several times a day	262	34.5 (1.5)	7/241	0.5 [0.2, 1.2
*Missing data*	*5*	-	*0/5*	-
Cereals:				
Never or almost never	4	34.7 (2.2)	0/4	NA
Not every day	162	34.4 (1.4)	5/148	Reference
Every day	488	34.4 (1.4)	24/428	2.6 [1.0, 6.9]
Several times a day	247	34.5 (1.4)	5/232	0.6 [0.2, 2.3]
*Missing data*	*92*	-	*0/10*	-
Dairy products:				
Never or almost never	7	33.1 (1.1)	2/4	Reference
Not every day	104	34.3 (1.3)	5/94	0.1 [0.0, 0.8]
Every day	395	34.4 (1.4)	14/356	0.1 [0.0, 0.5]
Several times a day	397	34.4 (1.5)	13/360	0.1 [0.0, 0.4]
*Missing data*	*8*	-	*0/8*	-
Meat:				
Never or almost never	22	34.0 (1.4)	1/20	NA
Not every day	336	34.5 (1.4)	11/302	Reference
Every day	449	34.4 (1.4)	16/409	1.1 [0.5, 2.3]
Several times a day	85	34.4 (1.6)	6/73	2.3 [0.8, 6.3]
*Missing*	*101*	-	*0/18*	-
**Dietary exposure (organic food intake)**				
Fruits and vegetables:				
Never	481	34.2 (1.4)	25/425	2.6 [1.1, 6.1]
Sometimes	340	34.6 (1.4)	7/312	Reference
Most of the time	85	34.4 (1.3)	2/80	NA
*Missing data*	*5*	-	*0/5*	-
Dairy products:				
Never	612	34.3 (1.4)	25/549	1.3 [0.3, 5.6]
Sometimes	231	34.6 (1.5)	7/210	1.0 [0.2, 4.7]
Most of the time	62	34.5 (1.3)	2/57	Reference
*Missing data*	*2*	-	*0/6*	-
Meat:				
Never	762	34.4 (1.4)	31/688	Reference
Sometimes	123	34.7 (1.5)	3/110	0.6 [0.2, 2.0]
Most of the time	17	34.1 (1.2)	0/15	NA
*Missing data*	*91*	-	*0/9*	-

HCB: head circumference at birth. SHCB: small head circumference at birth. NA: an odds ratio was not available because too few cases were observed (n<3).

In multivariate statistical models, no significant association was found between head circumference at birth or small head circumference on one hand and the variables describing the *in utero* exposure to pesticides on the other.

## Discussion

To the best of our knowledge, this study is the first one to simultaneously examine the association between all sources of *in utero* exposure to pesticides (i.e. occupational, domestic, environmental and dietary sources) and four common descriptors of fetal development (gestational age, birth weight, birth length and head circumference at birth). The prevalence of fetal growth restriction with respect to these parameters (i.e. prematurity, low birth weight, low birth length and small head circumference) was also considered.

Fetal growth is conditioned by many different environmental, genetic, metabolic, nutritional and placental factors. It is well known that adverse fetal growth is a good predictor of neonatal mortality and morbidity [17]–[19]. Fetal growth restriction may therefore be a determining factor for some diseases of childhood (since infants with poor head growth appear to run an increased risk of cerebral palsy, cognitive impairment and behavioral disorders) [20], [21] (ii) diseases of adolescence (since extremely low birth weight may be associated with a higher prevalence of developmental delay, neurosensory impairments (seizures, visual problems), learning disabilities and hyperactivity) and diseases of adulthood (hypertension, diabetes and hyperlipidemia) [20], [21]. Unfortunately, these factors are only seldom analyzed in the literature.

The inconsistency of the literature results may be explained (at least in part) by the fact that studies evaluating the association between pesticide exposure and fetal growth have used several different definitions of low birth weight. In fact, studies considering the proportion of small-for-gestational-age infants generally consider the 10^th^ percentile and include only the gestational age (plus, in some cases, the baby's gender) as an adjustment parameter. In contrast, our study low birth weight or length as being below the 5^th^ percentile after adjustment for various maternal and neonatal characteristics (the mother's age, weight, and height, the rank of birth, the gestational age and the baby's gender), as recommended by the AUDIPOG study [15]–[16].

In the present study, we considered almost all the confounding factors known or suspected in the literature to have an effect on fetal development. However, some of these factors (such as the consumption of alcohol and/or illicit drugs during pregnancy) could not be analyzed further because of the low number of affirmative replies. For ethical reasons, we chose not to probe a number of other factors (such as the parent's financial situation and ethnic origin); this might constitute a source of study bias. Moreover, the fact that pesticide levels were not measured in this study might constitute another source of bias; however, data obtained from maternal questionnaires were used as a proxy for pesticide exposure.

### 4.1 Occupational exposure to pesticides

Our results revealed that 4.3% of the mothers and 8.7% of the fathers had an occupation that potentially involved pesticide exposure. We found that 1.5% of the mothers and 4.2% of the fathers worked in agriculture (i.e. 2.9% of all parents). The literature studies differ in terms of their locations, study populations, exposure assessment methods and fetal parameters - making it difficult to compare the respective results. Our results highlighted an association between self-reported maternal occupational exposure to pesticides during pregnancy and the risk of low birth weight. We also found an association between estimated paternal occupational exposure to pesticides on one hand and low gestational age and the risk of the prematurity on the other. However, it is important to note that our cohort was recruited from the general population, in which only some of the parents were occupationally exposed to pesticides. This situation contrasts with literature studies of specifically exposed cohorts of farmers or other agricultural workers. The retrospective study of the general population performed in the USA by Savitz et al. [3] found an association between small-for-gestational-age status and self-reported occupational parental exposure to pesticides. In Colombia, the study by Restrepo et al. [5] revealed a moderately strong relationship between an increased risk of prematurity and reported occupational exposure to pesticides among female workers and the wives of male workers in floriculture. In contrast, the study in agricultural areas in Norway by Kristensen et al. [4] found that the rates of prematurity and small-for-gestational-age status were lower for farmers than for non-farmers. In the latter study, pesticide exposure was assessed by means of national census data on indicators such as farm size, the number and types of livestock, and so on.

Despite some small discrepancies between the above-mentioned results, there appears to be a general consensus in which maternal and/or paternal occupational exposure to pesticides is associated with adverse effects on fetal development and adverse birth outcomes.

### 4.2 Domestic exposure to pesticides

Our study also considered domestic pesticides but did not focus on any one substance or group of substances in particular (in contrast to several literature studies). We found that self-reported maternal exposure to pesticides for houseplants was correlated with lower birth weight. In contrast, we did not find any association between maternal exposure to pesticides for outdoor plants and poor fetal growth. Our findings are consistent with Petit et al.'s [14] results on insecticide use on indoor plants in the Brittany region of western France but not with their data on use on garden plants. In fact, Petit et al.'s retrospective study found that higher self-reported *in utero* exposure to household insecticides for use on plants (and especially outdoor plants) was related to lower birth weight and head circumference [14].

We did not find any association between fetal growth and maternal exposure to domestic insecticides. Our findings are consistent with those of Petit et al. [14] regarding the association between residential use of insect control on one hand and birth weight and head circumference at birth on the other hand. Similarly, our results are in line with the reports by Berkowitz et al. [8] and Whyatt et al. [9] in the USA, which did not find any association between self-reported use of domestic pesticides, prenatal personal ambient air or umbilical cord blood levels of some domestic insecticides on one hand and birth weight, birth length or head circumference at birth on the other. The questionnaire used in these two studies of the same cohort probed domestic use of pesticides against three groups of pests only (cockroaches, rodents and others). In the retrospective study performed by Savitz et al. [3] in the USA, self-reported exposure to household pesticides was associated with the risk of small-for-gestational-age status but not with prematurity. The questionnaire used in Savitz et al.'s study [1] probed exposure very generally via a single question on whether or not the mother and/or the father had been exposed to household pesticides.

In the present study, we found that maternal exposure to veterinary antiparasitics was associated with a lower birth weight. To the best of our knowledge, our study is the first to have assessed the relationship between maternal exposure to veterinary antiparasitics and fetal growth. In contrast, we did not find any association between fetal growth and maternal exposure to human antiparasitics.

### 4.3 Environmental exposure to pesticides

In our questionnaire, environmental exposure was assessed as self-reported proximity (within 1 km) of the mother's home during pregnancy to areas in which pesticides are usually widely applied (crop fields, green areas and transportation networks (highways, railroads and airports)). We did not observed any association between fetal growth and this proximity, in agreement with the results of the study by Petit et al. [14] in France; the latter researchers did not find any association between agricultural activities in the mother's place of residence during early pregnancy (based on data on agricultural activities from the national census) and birth weight (as a continuous variable) or the risk of low birth weight. In contrast, Xiang et al.'s [6] study in the USA (based on remote sensing and a geographic information system (GIS)) found an association between low birth weight and total crop production area within a 300 m buffer zone around the mother's residence. However, Xiang et al. [6] did not examine fetal growth parameters other than low birth weight. However, it is possible that the mothers in the present MecoExpo study (especially those living on the outskirts of cities) may have over-estimated the distance between their residence and crop fields.

### 4.4 Dietary exposure to pesticides

We did not find a significant association between fetal growth on one hand and overall or organic food intake on the other. Our questionnaire asked the mother about her eating behavior during a period (pregnancy) in which the diet is very likely to change. We cannot rule out the presence of recall bias, since the questionnaire was completed after delivery.

In conclusion, our present results demonstrate that both maternal and paternal occupational exposure to pesticides during pregnancy is associated with low gestational age and a greater risk of prematurity and low birth weight. Maternal domestic exposure to pesticides for houseplants and to veterinary antiparasitics was associated with low birth weight. Our questionnaire served as a wide-ranging tool for characterizing all the possible sources of prenatal exposure to pesticides. In the future, we could better characterize the exposure pathways and the nature of the compounds involved by complementing our questionnaire-based data with data generated by tools such as GISs and environmental or biological assays of pesticide residues.
